# Contrasting patterns of bacterial communities in the rearing water and gut of *Penaeus vannamei* in response to exogenous glucose addition

**DOI:** 10.1007/s42995-021-00124-9

**Published:** 2022-01-01

**Authors:** Lei Huang, Haipeng Guo, Zidan Liu, Chen Chen, Kai Wang, Xiaolin Huang, Wei Chen, Yueyue Zhu, Mengchen Yan, Demin Zhang

**Affiliations:** 1 State Key Laboratory for Managing Biotic and Chemical Threats to the Quality and Safety of Agro-Products, Ningbo University, Ningbo, 315211 China; 2 School of Marine Sciences, Ningbo University, Ningbo, 315211 China; 3 Zhejiang Institute of Freshwater Fisheries, Huzhou, 313001 China; 4 Zhejiang Mariculture Research Institute, Wenzhou, 325005 China

**Keywords:** Glucose addition, C/N ratio, Shrimp, Gut microbiota, Bacterioplankton community

## Abstract

Supplementing exogenous carbon sources is a practical approach to improving shrimp health by manipulating the microbial communities of aquaculture systems. However, little is known about the microbiological processes and mechanisms of these systems. Here, the effects of glucose addition on shrimp growth performance and bacterial communities of the rearing water and the shrimp gut were investigated to address this knowledge gap. The results showed that glucose addition significantly improved the growth and survival of shrimp. Although the α-diversity indices of both bacterioplankton communities and gut microbiota were significantly decreased by adding glucose, both bacterial communities exhibited divergent response patterns to glucose addition. Glucose addition induced a dispersive bacterioplankton community but a more stable gut bacterial community. Bacterial taxa belonging to *Ruegeria* were significantly enriched by glucose in the guts, especially the operational taxonomic unit 2575 (OTU2575), which showed the highest relative importance to the survival rate and individual weight of shrimp, with the values of 43.8 and 40.6%, respectively. In addition, glucose addition increased the complexity of interspecies interactions within gut bacterial communities and the network nodes from Rhodobacteraceae accounted for higher proportions and linked more with the nodes from other taxa in the glucose addition group than that in control. These findings suggest that glucose addition may provide a more stable gut microbiota for shrimp by increasing the abundance of certain bacterial taxa, such as *Ruegeria*.

**Supplementary Information:**

The online version contains supplementary material available at 10.1007/s42995-021-00124-9.

## Introduction

The Pacific white shrimp (*Penaeus vannamei*), the most productive cultured shrimp, is being threatened by the spread of several *Vibrio*-associated diseases, such as early mortality syndrome (EMS) (Lightner et al. 2012) and acute hepatopancreatic necrosis disease (AHPND) (Lee et al. 2015), which together cause a massive drop in global shrimp production (Huang et al. 2020a). To address aquaculture disease problems, many efforts have been made to explore new farming methods, of which the biofloc technology (BFT) is one of the most successful examples. BFT is a practical and easy-to-use aquaculture approach, that works by supplementing carbon sources to achieve a C/N ratio of more than 10:1 in the rearing water (Avnimelech 1999). Ample studies have demonstrated that adding carbon sources (e.g., molasses and glucose) can improve water quality and enhance the growth and disease resistance of shrimp (Hostins et al. 2019; Panigrahi et al. 2018). However, the probiotic mechanisms of this technique are still elusive.

As is well known, supplementing carbon sources to aquaculture systems is a microbial management strategy. Continuous addition of carbohydrates will induce an assemblage of heterotrophic bacteria, which can assimilate inorganic nitrogen and convert it into microbial protein, thus not only improving the water quality but also providing necessary nutrients for the growth of shrimp (Nevejan et al. 2018). Importantly, heterotrophic bacteria can establish stable and antagonistic bacterial communities, which help protect shrimp from external pathogens (Crab et al. 2010; Hostins et al. 2019). Previous studies have demonstrated that increasing the C/N ratio of the inputs in aquaculture systems by adding additional carbon sources, such as glucose (Zheng et al. 2018), molasses (Panigrahi et al. 2018), sucrose (Zhu et al. 2021) and enzyme-hydrolyzed cassava dregs (Shang et al. 2018), can reduce inorganic nitrogen levels and shift the bacterioplankton community composition. Qiao et al. (2017) demonstrated that in low salinity culture systems *P. vannamei* fed glucose had a better growth performance and a higher relative abundance of *Actinobacteria* in the intestines than shrimp fed corn starch. It has also been reported that supplementing carbon sources (glucose and sucrose) benefited the growth of some potential probiotics in the rearing water, such as *Bacillus* (Zheng et al. 2018) and Rhodobacteraceae (Zhu et al. 2021). These planktonic bacteria induced by carbon sources will inevitably affect the gut microbiota of shrimp because aquatic animals are constantly and directly in contact with the surrounding water. However, an intriguing scientific question as to whether the response pattern of gut microbiota to the added carbohydrate is the same as that of planktonic bacteria remains unclear.

Gut microbiota are sometimes considered to be another “brain” of the host, and they play an irreplaceable role in host nutrient acquisition (Sullam et al. 2015), immune response (Qu et al. 2021), and pathogen defense (Libertucci and Young 2019). Previous studies have shown a tight link between gut microbiota and shrimp health (Cornejo-Granados et al. 2017; Xiong et al. 2015). It has been reported that outbreaks of *Vibrio*-related diseases, such as white faeces syndrome and AHPND, are closely associated with the gut microbiota dysbiosis (Dong et al. 2021; Huang et al. 2020b). The assemblage of probiotic bacteria in the gut helps stabilize the gut microbiota through preventing the colonization and overgrowth of pathogens, thus helping to maintain animals’ health. Previous studies have widely reported that adding carbohydrates to the shrimp culture system can significantly increase the accumulation of certain potential probiotic bacteria in the gut, such as bacteria from Rhodobacteraceae (Guo et al. 2020), which has been reported to be positively correlated with the growth performance (Huang et al. 2020a), pathogen defense (Yao et al. 2018) and cold resistance (Liu et al. 2019). However, the probiotic effects of these bacteria enriched by adding carbohydrates remains to be clarified.

Here, we hypothesize that glucose-mediated improvement of shrimp health was associated with a change in the bacterioplankton communities and gut microbiota. To test this hypothesis, the effects of supplementing glucose on bacterial communities of shrimp culture system were investigated. We first compared the response patterns of bacterioplankton communities and gut microbiota to glucose addition, and then the effect of glucose on the homeostasis of gut microbiota was investigated. Finally, the key potential probiotics enriched by adding glucose were identified, and their relative importance in promoting the growth performance of shrimp were preliminarily evaluated.

## Results

### Water quality and shrimp performance

Water temperature and dissolved oxygen concentration strongly fluctuated during the experiment, while nitrite, phosphate and total phosphorus showed a stable increasing trend over the culture time (Supplementary Fig. S1). In the late culture stage, the ammonium concentration (Day 14, 15.0 ± 3.4 mg/L; Day 21, 1.6 ± 2.0 mg/L) and pH value (Day 17, 8.1 ± 0.2; Day 21, 7.0 ± 0.4) decreased sharply, while the concentration of nitrite in both the control and glucose addition groups increased to more than 28 mg/L at Day 21. Although the concentrations of ammonium (Day 14), nitrite (Days 14 and 21), phosphate (Days 7, 14 and 21) and total phosphorus (Day 21) in the glucose addition group were significantly lower after the addition of glucose (Independent *t*-test, *P* < 0.05), compared to the control group, they were still high in the late culture stage (e.g., the ammonium concentration at Day 14 was 13.2 ± 3.0 mg/L). After 21 days of culture, the yield, survival rate, individual weight and individual length of shrimp in the glucose addition group were significantly higher than those in the control group (Independent *t*-test, *P* < 0.01), with increment rates of 132.2%, 112.4%, 21.8% and 8.2%, respectively (Supplementary Fig. S2).

### Differences in bacterial community diversity and structure

**Fig. 1:**
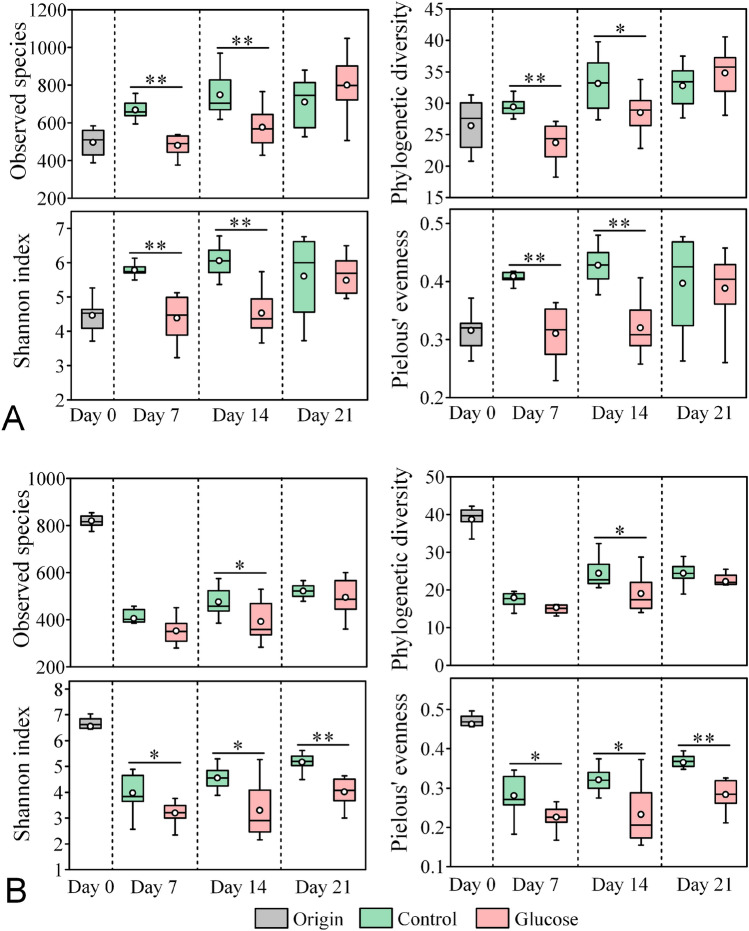
Dynamics of bacterial ɑ-diversity in the rearing water (**A**) and shrimp guts (**B**). Origin: original bacterial communities of the water or gut samples. Data are presented as means ± standard errors (*n* = 8). The statistical significance of the differences between the two groups are tested using an Independent *t*-test (*P < 0.05, **P < 0.01)

In the rearing water, the α-diversity indices, including observed species, phylogenetic diversity, Shannon index, and Pielou's evenness, were significantly lower in the glucose addition group than those in the control at Days 7 and 14 (Independent *t*-test, *P* < 0.05) (Fig. 1A). In gut samples, all these indices in both groups declined sharply in the early culture stage (from Day 0 to Day 7). Consistent with the water samples, all the diversity indices (except the observed species and phylogenetic diversity at Days 7 and 21) of the glucose addition group were significantly lower than those of the control at each sampling day (Independent *t*-test, *P* < 0.05) (Fig. 1B).

**Fig. 2:**
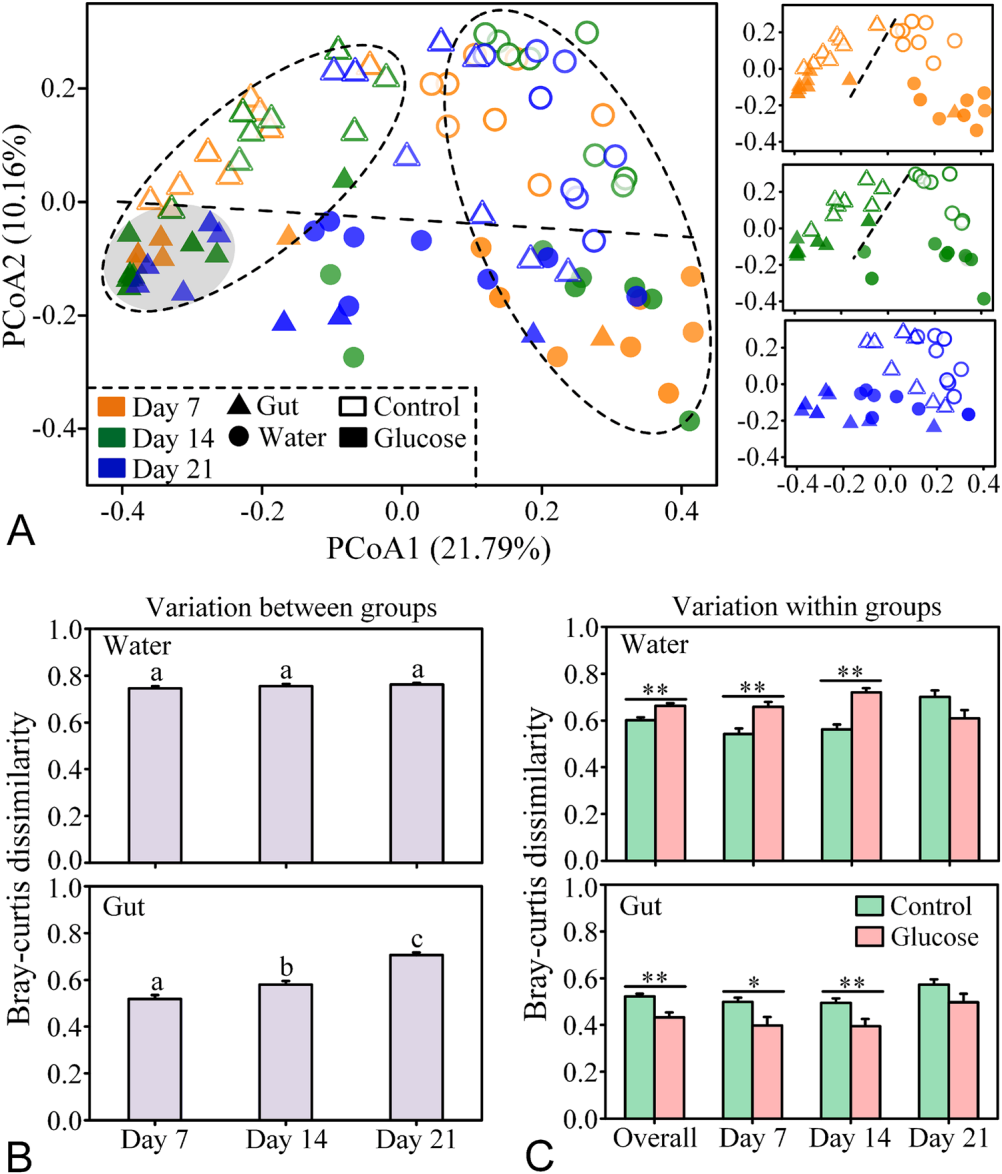
**A** Principal Coordinate Analysis (PCoA) plot based on the Bray–Curtis dissimilarity show the dissimilarities of bacterial community between the control and glucose groups in the water and gut samples. Original (Day 0) samples are deviated far from the samples at other time points, so they are not shown in the figure. The gray shadow indicates that the intestinal bacterial communities of the glucose group are very similar. **B** Bray–Curtis dissimilarity analysis reveal the dissimilarities of parallel samples between the two groups in rearing water and shrimp guts. Different lowercase letters on the histogram indicate a significant difference (one-way ANOVA, *P* < 0.05). **C** Bray–Curtis dissimilarity analysis reveal the similarities of parallel samples within the control or glucose addition groups. The statistical significance of the differences between the two groups are tested using an Independent *t*-test (*P < 0.05, **P < 0.01)

The PCoA plot showed a clear difference in bacterial community composition (BCC) between the control and glucose addition groups, in both the rearing water and the shrimp gut (Fig. 2A). This pattern was further corroborated by the ANOSIM and PERMANOVA analyses, which indicated that the BCCs of the water and the gut samples were significantly different between the two groups at each sampling point (*P* < 0.05) (Table 1). Although glucose addition changed the BCCs of both the rearing water and the shrimp gut, their response patterns were markedly different. The bacterioplankton communities showed strong changes once the glucose was added, and these differences in BCCs between the control and glucose addition groups were maintained throughout the experiment (Fig. 2B). In contrast, the dissimilarities in gut microbiota between the two groups were relatively small at Day 7, while this difference gradually increased over culture time (Fig. 2B). In addition, the dissimilarities in gut microbiota within the glucose addition group at Days 7 and 14 were significantly lower than those within the control group (Independent *t*-test, *P* < 0.05), while an opposite trend was observed in the water samples (Fig. 2C).

**Table 1:** Analysis of similarity (ANOSIM) and permutational multivariate analysis of variance (PERMANOVA) based on Bray–Curtis dissimilarity are applied to evaluate the differences in bacterial community compositions of the rearing water and shrimp guts between the control and glucose addition groups at each sampling day

		ANOSIM		PERMANOVA	
		R	*P*	F	*P*
Water	Global	0.386	0.001	7.283	0.001
	Day 7	0.677	0.001	4.881	0.001
	Day 14	0.523	0.001	3.759	0.001
	Day 21	0.494	0.001	3.540	0.001
Gut	Global	0.327	0.001	9.966	0.001
	Day 7	0.203	0.011	2.127	0.024
	Day 14	0.522	0.001	5.865	0.001
	Day 21	0.605	0.001	5.926	0.004

### Differences in bacterial community compositions

The main dominant phyla (as well as proteobacterial classes, > 1% at least in one group at any sampling time) in the rearing water were Alphaproteobacteria, Bacteroidetes and Gammaproteobacteria, with average relative abundances of 38.9, 28.2 and 18.6%, respectively (Supplementary Fig. S3A); the main dominant families (> 3% at least in one group at any sampling time) were Rhodobacteraceae, Flavobacteriaceae, Saprospiraceae and Vibrionaceae, accounting for 32.1, 9.7, 8.5 and 6.9%, respectively. The relative abundances of Vibrionaceae (14.9% vs 1.0%) in the glucose addition group were significantly higher (Independent *t*-test, *P* < 0.05) than those in the control, while the relative abundance of Flavobacteriaceae in the control group (15.8%) was significantly higher (*P* < 0.05) than that in the glucose addition group (5.8%) (Supplementary Fig. S3A). In the shrimp gut, the most abundant class and family were Alphaproteobacteria (58.0%) and Rhodobacteraceae (54.2%), respectively (Supplementary Fig. S3B). The relative abundances of Rhodobacteraceae were significantly higher (*P* < 0.05) in the glucose addition group than those in the control at Days 14 (73.5% vs. 53.8%) and 21 (56.1% vs. 37.7%) (Supplementary Fig. S3B).

**Fig. 3:**
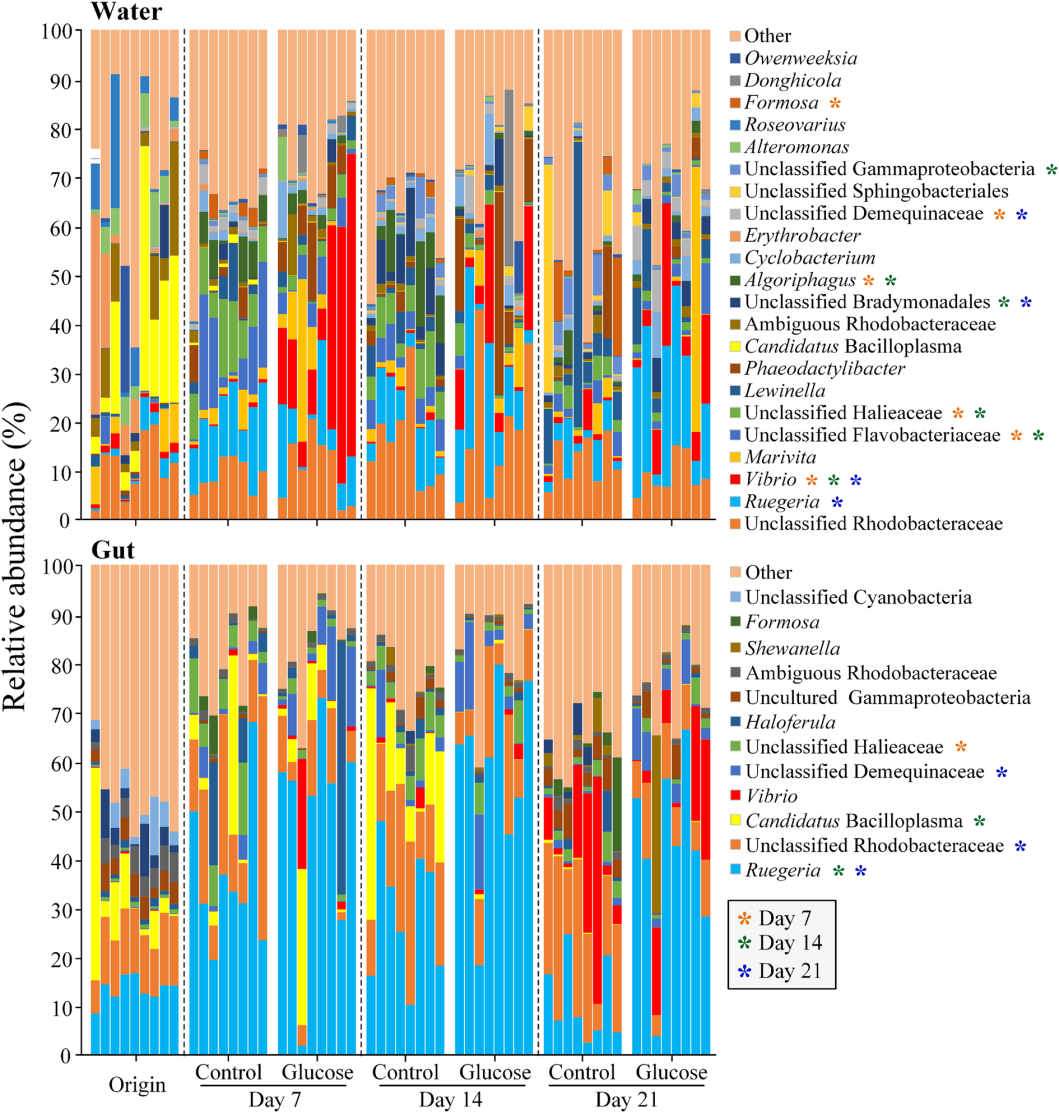
Relative abundances of the dominant genera (average relative abundance > 3% at least in one group at any sampling time) in the rearing water and shrimp guts. The orange, green and blue asterisks indicate the significant differences (Independent *t*-test, *P* < 0.05) between the control and glucose addition groups at Days 7, 14, and 21, respectively

At the genus level, the composition of the bacterioplankton communities was very diverse, mainly including unclassified Rhodobacteraceae (average relative abundance of 12.3%), *Rugeria* (9.9%) and *Vibrio* (6.9%), while the most abundant genus in the shrimp gut was *Rugeria* (33.4%), followed by unclassified Rhodobacteraceae (14.5%), *Candidatus* Bacilloplasma (5.5%) and *Vibrio* (4.0%) (Fig. 3). In the rearing water, the relative abundances of *Vibrio* (Day 7, 25.8% vs. 0.5%; Day 14, 9.6% vs. 0.6%; Day 21, 9.0% vs. 1.9%) and *Rugeria* (Day 21, 19.9% vs. 4.7%) were significantly higher (*P* < 0.05) in the glucose addition group than those in the control group; while the unclassified Flavobacteriaceae (Day 7, 3.6% vs. 10.9%; Day 14, 0.5% vs. 5.0%), unclassified Halieaceae (Day 7, 2.3% vs. 9.5%; Day 14, 2.0% vs. 6.8%) and *Algoriphagus* (Day 7, 1.4% vs. 4.6%; Day 14, 0.4% vs. 6.3%) had higher relative abundances in the control than in the glucose addition group (Fig. 3). In the gut samples, the relative abundances of *Ruegeria* (Day 14, 57.5% vs. 28.6%; Day 21, 41.4% vs. 10.9%) and unclassified Demequinaceae (Day 21, 5.0% vs. 0.7%) were significantly higher, and the relative abundances of unclassified Rhodobacteraceae (Day 21, 9.1% vs. 20.5%) and *Candidatus* Bacilloplasma (Day 14, 0.2% vs. 14.7%) were lower in the glucose addition group compared with those in the control (Fig. 3).

**Fig. 4:**
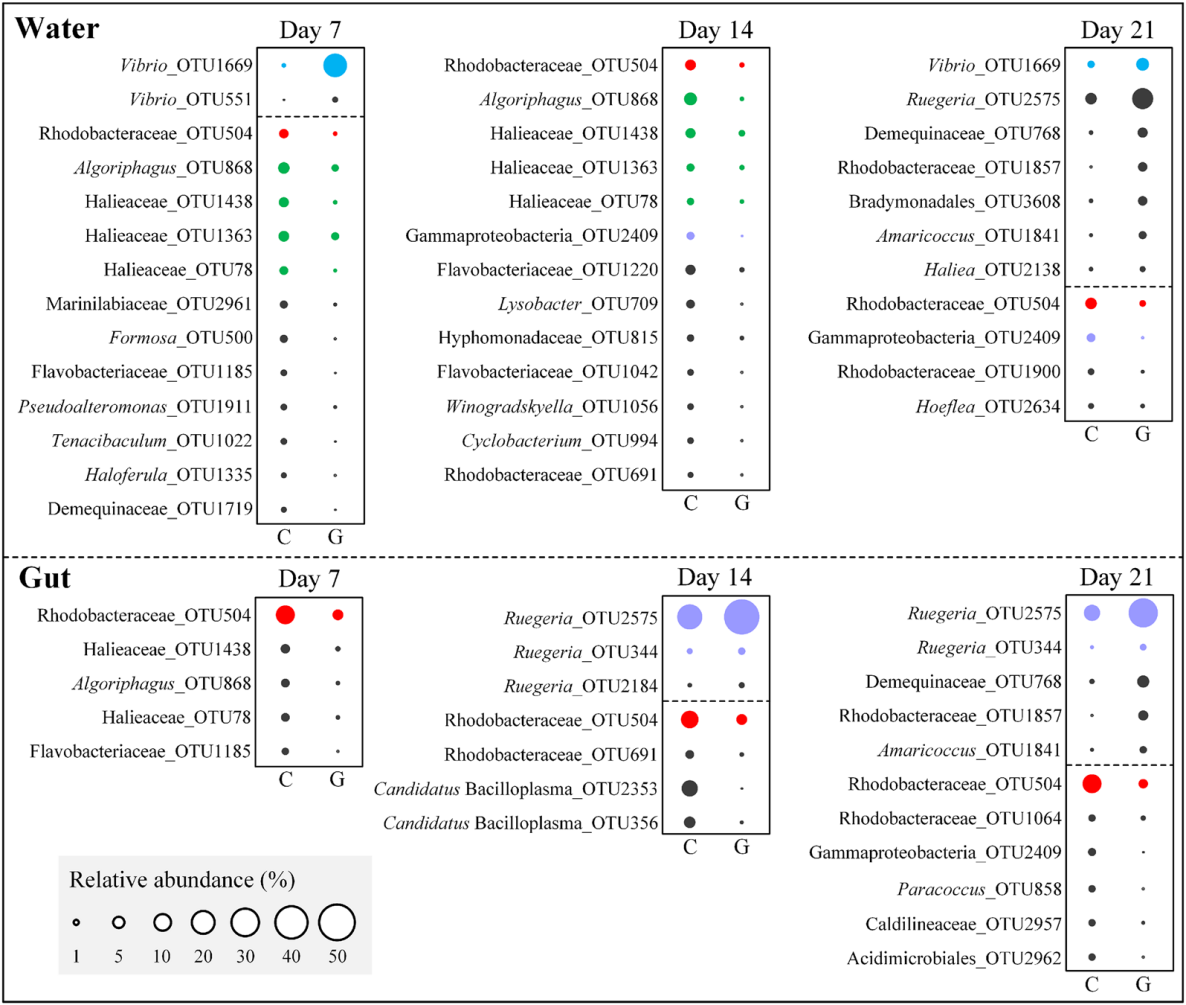
Bubble plots show the abundant OTUs (average relative abundance > 1%) with significant differences (Independent *t*-test, *P* < 0.05) between the control and glucose groups in the rearing water and shrimp guts at each sampling day. Red bubbles indicates that they appear at all sampling points; Green, blue and purple bubbles indicates that they appeared at Days 7 and 14, Days 7 and 21, and Days 14 and 21, respectively. The relative abundances of OTUs were square root transformed and showed with the size of bubbles

The discriminatory OTUs with significant differences (Independent *t*-test, *P* < 0.05) between the control and the glucose addition groups at least at one sampling time point were further screened (Fig. 4). In the rearing water, a total of 30 distinct OTUs were identified, of which 8 were enriched OTUs and 21 were depleted OTUs by glucose addition. The enriched OTUs mainly consisted of two *Vibrio* OTUs (OTU1669 and 551) at Day 7, and one *Vibrio* OTU1669, three Rhodobacteraceae OTUs (OTU2575, 768 and 1857), and three other OTUs (OTU3608, 1841 and 2138) at Day 21. Of the depleted OTUs, one Rhodobacteraceae OTU504 was shared by all sampling days, four OTUs (OTU868, 1438, 1363 and 78) shared by Days 7 and 14, and one Gammaproteobacteria OTU2409 shared by Days 14 and 21 (Fig. 4). In the shrimp gut, six OTUs were significantly enriched by glucose addition, these included two *Ruegeria* OTUs (OTU344 and 2575) shared by Days 14 and 21, one *Ruegeria* OTU2184 at Day 14 and three OTUs (OTU768, 1841 and 1857) at Day 21; of the 14 OTUs depleted by glucose addition, Rhodobacteraceae OTU504 shared by all sampling days, and four, three and six OTUs only appeared at Days 7, 14 and 21, respectively (Fig. 4).

### Relationships between discriminatory OTUs and shrimp growth parameters

**Fig. 5:**
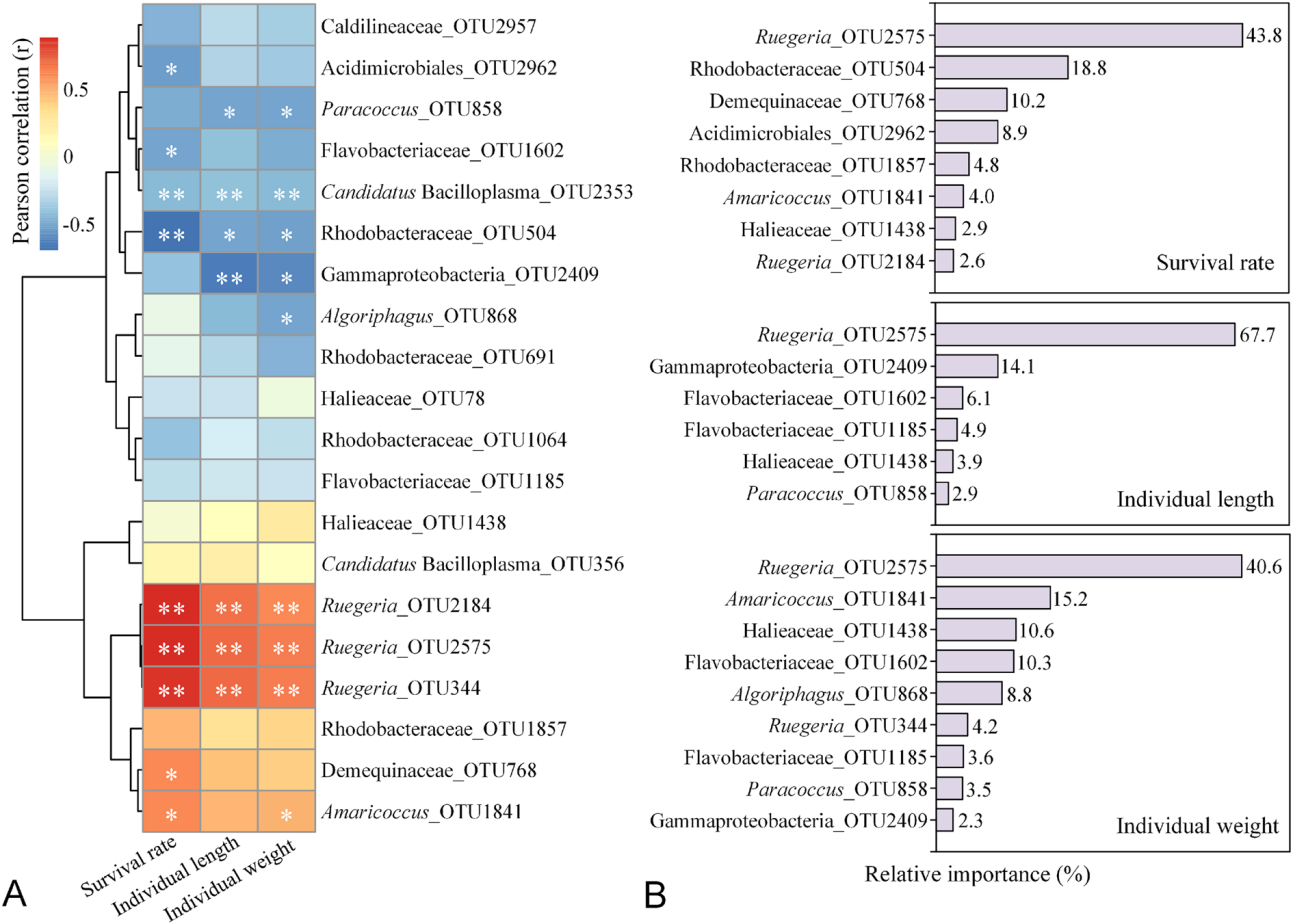
Heatmaps illustrate the relationship (Pearson’ correlation) between the discriminatory OTUs of the shrimp guts (from Fig. 4) and shrimp growth parameters at Day 21. * and ** represent statistical significance at *P* < 0.05 and *P* < 0.01 levels, respectively. The relative importance of these discriminatory OTUs in the shrimp guts on shrimp growth parameters in the BRT model. OTUs with relative importance greater than 2% are displayed in the figure

Correlations of the discriminatory OTUs with the survival rate, individual lengths and individual weights of shrimp were investigated (Fig. 5A and Supplementary Fig. S4A). In the shrimp gut, *Ruegeria* OTUs (344, 2184 and 2575), *Amaricoccus* OTU1841 and Demequinaceae OTU768 exhibited significant and positive correlations (Pearson’s correlation, *P* < 0.05) with most growth parameters, while *Candidatus* Bacilloplasma OTU2353, Rhodobacteraceae OTU504 and Gammaproteobacteria OTU2409 showed an opposite trend (Fig. 5A). In the rearing water, *Ruegeria* OTU2575, Rhodobacteraceae OTU1857, *Pseudoalteromonas* OTU1911, Demequinaceae OTU768, *Haliea* OTU2138 and Bradymonadales OTU3608 had significant and positive correlations (*P* < 0.05) with most growth parameters, while Rhodobacteraceae OTUs (504 and 1900), Gammaproteobacteria OTU2409, *Hoeflea* OTU2634 and *Formosa* OTU500 exhibited significant and negative correlations (*P* < 0.05) with some growth parameters (Supplementary Fig. S4A).

To discern the relative importance of the above discriminatory OTUs on the growth performance of shrimp, a BRT modeling analysis combined with statistical and machine learning techniques was performed (Fig. 5B and Supplementary Fig. S4B). In the shrimp gut, *Ruegeria* OTU2575 was the most important variable on survival rate (43.8%), individual length (67.7%) and individual weight (40.6%) of shrimp; Rhodobacteraceae OTU504 (18.8%) was the second most important variable on survival rate, followed by Demequinaceae OTU768 (10.2%) and Acidimicrobiales OTU2962 (8.9%). Gammaproteobacteria OTU2409 accounted for 14.1% the relative importance on individual length, and *Amaricoccus* OTU1841, Halieaceae OTU1438 and *Algoriphagus* OTU868 accounted for 15.2, 10.6, 10.3 and 8.8% of the relative importance on individual weight, respectively (Fig. 5B). In the rearing water, *Amaricoccus* OTU1841 (51.3%) and Rhodobacteraceae OTU504 (30.1%) accounted for more than 80% of the relative importance on survival rate; Demequinaceae OTU768, *Ruegeria* OTU2575 and Rhodobacteraceae OTU1857 accounted for 40.7, 27.2 and 11.2% for the individual length, and 30.2, 23.0 and 18.2% for the individual weight, respectively (Supplementary Fig. S4B).

### Effects of glucose addition on the co-occurrence networks of bacterial communities

**Fig. 6:**
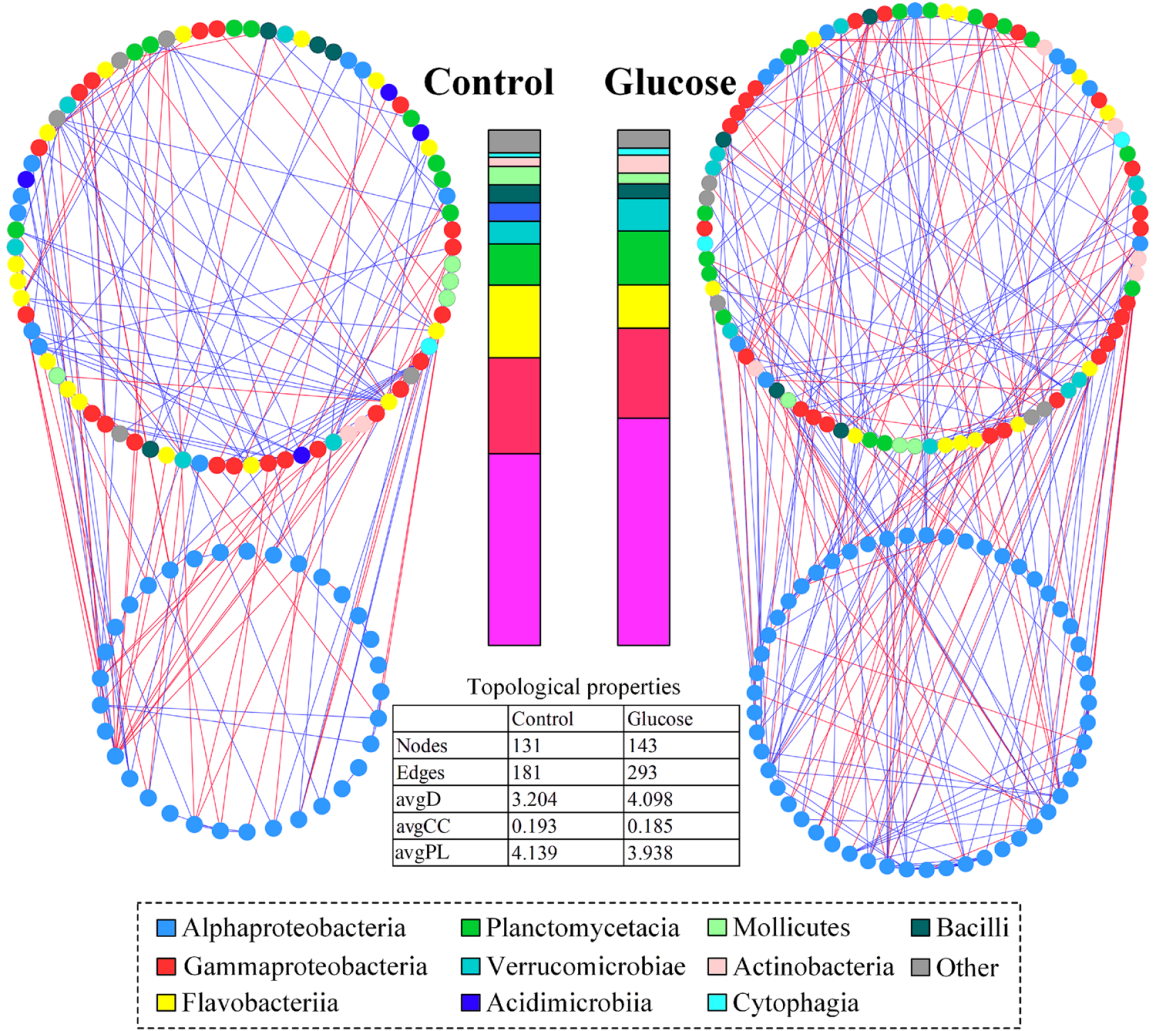
Networks and related topological properties of the control and glucose groups in shrimp guts. The color of the node shows different dominant classes, and the relative abundances of these classes are showed in the bar charts. The color of the edge shows positive (green) or negative (red) correlations between nodes. Rhodobacteraceae (blue circles) is separately grouped in each network. *avgD* average degree, *avgCC* average clustering coefficient, *avgPL* average path length

To evaluate the effects of glucose addition on the interspecies interactions of gut microbiota and bacterioplankton communities, molecular ecological networks (MENs) of the control and glucose addition groups were constructed using an RMT-based network inference approach (Fig. 6 and Supplementary Fig. S5). The network connectivity distribution curves of the control and glucose addition groups had comparable similarity thresholds (0.760 and 0.710 in guts, 0.780 and 0.770 in water, respectively) when plotted and fitted with the power law model, suggesting that the constructed networks were scale-free. The co-occurrence networks of gut microbiota were more complex and better connected with a higher average degree and lower average path distance in the glucose addition group than those of the control (Fig. 6), while the overall network complexity of the bacterioplankton communities of the two groups were similar (Supplementary Fig. S5). Notably, the proportions of the node belonging to Rhodobacteraceae were higher in the glucose addition group than in control (gut: 37.1% vs. 25.2%; rearing water: 30.0% vs. 14.2%), and these Rhodobacteraceae OTUs also contributed more links to other non- Rhodobacteraceae nodes (gut: 58.0% vs. 42.0%; rearing water: 49.7% vs. 12.9%) (Fig. 6, Supplementary Fig. S5, Supplementary Table S1).

## Discussion

### Effects of glucose addition on water quality and shrimp growth performance

Increasing the C/N ratio of inputs to aquaculture systems by supplementing exogenous carbon sources has been considered an effective method to improve water quality (Panigrahi et al. 2018; Shang et al. 2018; Xu et al. 2016). Consistent with this concept, this study also found that adding glucose significantly reduced the concentrations of ammonium and nitrite in the rearing water (Supplementary Fig. S1). Even when the water environment was improved by adding glucose, the water quality condition in the late culture stage might still not have been conducive to the growth of shrimp. It is possible that the low rate of water exchange might have led to a deterioration of water quality in the late culture stage. Also, no bioflocs were formed during culture process, so the water purification capacity of the aquaculture ecosystem might have been insufficient. However, the addition of glucose still significantly improved the survival and growth of shrimp (Supplementary Fig. S2), indicating that the water quality improvement mediated by glucose addition might still be beneficial to promote the health of shrimp. To maintain good water quality conditions for aquaculture, we recommend inoculating bioflocs or probiotics in the water at the beginning of the breeding process.

### Glucose addition increased the stability of gut microbial community

It has been reported that an increase in the C/N ratio reduces microbial community diversity in lab-scale aquaculture biofilters (Michaud et al. 2014), and the application of exogenous glucose simplifies soil- and plant-derived microbiomes and thus decreases their bacterial community diversities (Goldford et al. 2018). In the present study, glucose addition did significantly decrease the bacterial α-diversity in both the rearing water and shrimp guts, especially at Days 7 and 14 (Fig. 1). One possible explanation for the decreased diversity is that glucose addition stimulated the rapid growth of some opportunistic taxa such as *Vibrio* and *Ruegeria*, thereby reducing the diversity of bacterial communities. Bacterial diversity has been considered an important indicator of community stability (Ives and Carpenter 2007; Shade et al. 2012), and it is generally accepted that higher bacterial diversity implies a more stable community with a stronger resistance to pathogen invasion (De Schryver and Vadstein 2014). Recently, ecologists have begun to question the relationships between diversity and stability, productivity and invasibility and have found that high bacterial diversity is not necessary for ‘better’ or ‘healthy’ ecological systems (Shade 2017). A recent human study found that there was no significant difference in the microbial diversity between healthy and diseased individuals, suggesting that the species diversity of human microbiomes is not a reliable indicator of disease (Ma et al. 2019). Consistently, Wang et al. (2019) reported that no significant differences were observed between healthy and WSSV-infected *P. vannamei*. Studies have even shown that the intestinal bacterial diversities of diseased shrimps were surprisingly higher than those of healthy shrimps (Cornejo-Granados et al. 2017; Zhou et al. 2019). In the current study, although glucose addition decreased the α-diversity of the bacterial communities in the rearing water and shrimp gut, the variability of the bacterial communities was increased in the water but decreased in the gut (Fig. 2C), further indicating that the higher α-diversity may not be enough to determine whether a bacterial community is more stable. Instead, the simplified bacterial community induced by glucose addition in the gut may possess more generalist core species which are better able to tolerate resource fluctuations (Kokou et al. 2019), and thus increase the stability of bacterial communities.

Interspecies interaction reflects the niche processes driving co-occurrence within biological communities. In recent years, an increasing number of studies have found that network complexity is strongly correlated with network stability (Ruan et al. 2020; Yuan et al. 2021). This study showed that glucose addition enhanced the complexity of the gut bacterial network (Fig. 6), suggesting that glucose addition might improve the stability of ecological networks in shrimp guts. Recent studies have shown that the complexity of interspecies interactions in the aquatic hosts was positively related to host health (Nie et al. 2017; Zhu et al. 2016). Zhu et al. (2016) demonstrated that shrimp disease reduced the complexity and cooperative activities of species-to-species interactions. Similarly, another study showed that ayu (*Plecoglossus altivelis*) infected with *V. anguillarum* had lower bacterial network complexity in guts compared with healthy controls (Nie et al. 2017). Consistent with these findings, the glucose addition group with better shrimp growth performance possessed a more complex gut bacterial network (Fig. 6, Supplementary Fig. S2), indicating that adding glucose might improve the health of shrimp by increasing the stability of the gut bacterial network. Notably, Rhodobacteraceae in the glucose addition group accounted for a much higher proportion in the gut bacterial networks, suggesting that Rhodobacteraceae might play an important role in improving network stability.

The homeostasis of gut microbiota is an important factor for sustaining host health. Multiple studies have illustrated that dysbiosis in gut microbiota contributes to several human diseases, such as inflammatory bowel disease (Zhang et al. 2018) and atherosclerotic cardiovascular disease (Jie et al. 2017). For shrimp, Yao et al. (2018) also demonstrated that the gut microbiota of healthy shrimps was more stable than that of diseased ones, suggesting that the homeostasis of gut microbiota is highly relevant to shrimp heath. Similarly, a recent study reported that the gut microbiota dysbiosis caused white feces syndrome (Huang et al. 2020b). In this study, glucose addition decreased the heterogeneity of gut microbiota (Fig. 2C) and improved the growth and survival of shrimp (Supplementary Fig. S2), suggesting that the increased stability of gut microbiota induced by adding glucose might contribute to shrimp health. Furthermore, the glucose addition group had a more closely-knit gut bacterial community structure (Fig. 2A), which is consistent with the ‘Anna Karenina principle’ for animal microbiomes that diseased microbial communities vary more in composition than healthy ones—paralleling Leo Tolstoy’s dictum that ‘all happy families are alike, but each unhappy family is unhappy in its own way’ (Zaneveld et al. 2017).

### Different response patterns of bacterioplankton community and gut microbiota to glucose addition

Previous studies demonstrated that supplementing carbon source, such as glucose (Wei et al. 2016), molasses (Cardona et al. 2016), and cassava flour (Shang et al. 2018), could affect the microbial community structure and composition of the rearing water and the shrimp gut in culture systems. Zheng et al. (2018) demonstrated that increasing the C/N ratio above 10:1 by adding glucose could significantly shift the structure and function of the bacterial communities in the *P. vannamei* culture system. In the current study, glucose addition also largely affected the microbial community assembly in shrimp aquaculture system; however, the BCCs of both the water and gut exhibited divergent response patterns to the exogenous carbon source. Bacterioplankton communities changed rapidly and dramatically with adding glucose, while the dissimilarities of gut bacterial communities caused by glucose addition increased over time (Fig. 2B, Table 1). Previous studies have reported that microbial community of water in ponds is directly affected by environmental factors (Hou et al. 2017), so the bacterioplankton communities will respond quickly once the physicochemical parameters are changed. Comparatively, bacterial communities of aquatic animals are influenced by not only their habitation environment (Tzeng et al. 2015) but also the host filtering (Schmidt et al. 2015), which might slow down the change of gut microbiota in the presence of glucose. The dissimilarity of gut microbiota between different treatments was extended with the increase of feeding time, indicating that the gut microbiota could be gradually changed by the continuous addition of glucose (Fig. 2B). The shrimp might ingest the bioflocs formed by regularly adding glucose in the rearing water at the later stage, and the microorganisms in bioflocs might affect the indigenous microbiota of shrimp gut (Huang et al. 2020a), thus increasing the difference of bacterial communities between control and glucose addition groups.

In the rearing water, glucose addition mainly increased the relative abundances of Vibrionaceae (e.g., *Vibrio*) and Rhodobacteraceae (e.g., *Ruegeria*), and decreased the relative abundances of Flavobacteriaceae and Halieaceae; while in the guts, glucose addition group enriched Rhodobacteraceae (e.g., *Ruegeria*) and suppressed the Mycoplasmataceae (e.g., *Candidatus* Bacilloplasma) (Figs. 3, 4, Supplementary Fig. S3). Consistent with this study, Shang et al. (2018) reported that adding carbon source (enzyme-hydrolyzed cassava dregs) increased the relative abundances of Rhodobacteraceae and Vibrionaceae, and reduced the relative abundance of Flavobacteriaceae in shrimp culture system. Notably, the relative abundances of Rhodobacteraceae (e.g., *Ruegeria*) in both the rearing water and shrimp guts were increased by glucose addition, but the increment was more obvious in the guts than in the rearing water (Fig. 3, Supplementary Fig. S3). Previous studies have reported that the *Roseobacter* clade, the main member of the Rhodobacteraceae, showed a high activity in glucose uptake in northwestern Mediterranean coastal waters (Alonso-Sáez and Gasol 2007), and was considered to dominate microbial glucose uptake in coastal North Sea waters (Alonso and Pernthaler 2006). It was reported that some Rhodobacteraceae species (e.g., *Ruegeria mobilis*) could form biofilms and produce certain secondary metabolites only when they were cultured statically or grown on solid surfaces (Porsby et al. 2008). The intestinal environment is more stable than the water environment, so it may be more conducive to the growth of Rhodobacteraceae. In contrast, the Vibrionaceae (e.g., *Vibrio*) in water rapidly bloomed after adding glucose, while the relative abundances of these taxa in the guts increased significantly at Day 21 (Fig. 3, Supplementary Fig. S3). Vibrionaceae is termed as fast-growing opportunistic* r-strategists*, and they can be stimulated to grow by the addition of glucose in seawater (Weinbauer et al. 2006). Thus, the rapid bloom of Vibrionaceae may be closely associated with glucose addition in water at the early stage. Although it has been well reported that gut microbiota of aquatic animals were affected to some extent by the microorganisms in the surrounding water (Xiong et al. 2018), we noted that the glucose-mediated the bloom of Vibrionaceae in water failed to increase their abundance in guts, but induced the accumulation of Rhodobacteraceae, especially its genus of *Ruegeria* in guts (Figs. 3, 4). Our previous study indicated that Vibrionaceae and Rhodobacteraceae showed an antagonistic relationship in the shrimp gut (Yao et al. 2018), which made us extremely suspect whether the lower *Vibrio* colonization in the presence of glucose was associated with the accumulation of *Ruegeria* in guts.

### Glucose-induced increase of *Ruegeria* in the gut might facilitate the health of shrimp

*Ruegeria*, the dominant genus of Rhodobacteraceae in both the water and guts, always had a higher relative abundance in the glucose addition group (Fig. 3), and the relative abundances of the dominant *Ruegeria* OTUs (2575, 344 and 1513) in the guts had a markedly positive correlation with growth traits and survival (Fig. 5A). *Ruegeria* is widely distributed in both the marine environment (Sonnenschein et al. 2017) and the gut of *P. vannamei* ( Wang et al. 2020; Zheng et al. 2017). Members of *Ruegeria* are symbiotic with aquatic hosts and these symbiosis effects may help the host to degrade carbohydrates in the gastrointestinal tract. Many *Ruegeria* species are also capable of synthesizing vitamin B12, which is essential for the growth of shrimp (Shiau and Lung 1993). These findings suggest that *Ruegeria* in the intestine of shrimp might play an important role in nutrient acquisition and absorption. Consistent with these observations, this study also found that *Ruegeria* OTU2575 in the gut contributed the most to the individual lengths and weights of the shrimp (Fig. 5B). In the rearing water also this taxa made a major contribution to explain these growth parameters (Supplementary Fig. S5B). These findings indicate that the glucose-induced increase of *Ruegeria* might help to promote the growth of shrimp.

Vibriosis caused by some *Vibrio* species, such as *V. harveyi*, *V. parahaemolyticus* and *V. anguillarum* etc., is regarded as one of the most prevalent diseases in shrimp and other aquaculture animals (Chatterjee and Haldar 2012; Ruwandeepika et al. 2012; Zhang et al. 2020). In this study, the abundance of *Vibrio* OTU1669 (the most abundant OTU of *Vibrio* in guts) showed a significant negative correlation with the survival rate of shrimp (data not shown), suggesting that the accumulation of *Vibrio* in the gut might be responsible for disease in shrimp. Interestingly, the three most abundant *Vibrio* OTUs showed stronger negative correlations with the three most abundant *Ruegeria* OTUs in guts than in water (Supplementary Fig. S6A), and the abundance of *Vibrio* OTU1669 increased in the rearing water rather than in guts after glucose addition (Fig. 4), indicating that *Ruegeria* might play a role in suppressing *Vibrio* in guts but not in water. Several studies have shown that some tropodithietic acid (TDA)-producing bacteria, such as *Ruegeria* spp., strongly inhibit the growth of several *Vibrio* spp., such as *V. anguillarum* and *V. coralliilyticus* ( Miura et al. 2019; Porsby et al. 2008). However, *Ruegeria* spp. only produce the anti-*Vibrio* compound TDA under stagnant growth condition and not in shaking broth cultures ( D'Alvise et al. 2014; Porsby et al. 2008). In this study, a *Ruegeria* strain was isolated from a shrimp gut, and this strain produced a brown pigment (probably TDA) that showed antibacterial activity against *Vibrio* spp. only in the stagnant culture environment (Supplementary Fig. S6B). This suggests that the gut might provide a stagnant environment for *Ruegeria* to produce antibacterial compounds; while the constantly agitated rearing water was not advantageous to producing these antibacterial substances. So, there was no antagonistic relationship between *Ruegeria* and *Vibrio* in the water. This result suggests that *Ruegeria*, as a probiotic, should be fed to aquatic animals instead of adding them to the water, but this needs to be verified in future studies.

## Conclusions

In summary, glucose addition altered the diversity, structure and composition of bacterioplankton communities and gut microbiota, although the responses of each differed. Glucose addition increased the variation of bacterioplankton communities, but improved the stability of gut bacterial communities. Glucose addition mainly promoted the growth of *Ruegeria* OTU2575 in guts, which had the highest relative importance to survival rates and individual lengths and weights of the shrimp. Furthermore, a co-occurrence network analysis showed that Rhodobacteraceae members enriched by glucose addition helped to enhance the bacterial network stability. These discoveries will provide potential ideas and a theoretical basis for understanding the microbiological mechanism of adding glucose to improve shrimp growth performance. Further work is needed to confirm these findings.

## Materials and methods

### Experimental design and sampling

This experiment was conducted at Zhejiang Mariculture Research Institute in Wenzhou, Zhejiang Province, China (27°51′26.28″ N, 120°50′0.76″ E). Before the experiment, 16 indoor plastic tanks (600 L) were filled with 400 L sand-filtered seawater, and then randomly stocked with 150 healthy shrimps (length, 6.13 ± 0.64 cm; weight, 3.03 ± 0.80 g) obtained from Zhejiang Mariculture Research Institute. Eight tanks were randomly assigned as the glucose addition group, and the remaining eight were used as the control group without glucose addition. Glucose comprising 65% of the feed was added to the experimental group to achieve the desired input C/N ratio of 10:1. The C/N ratios of the input were precisely calculated based on the carbon–nitrogen contents of the feed (44.20% w/w carbon, 7.04% w/w nitrogen; Alpha Feed Co., Ltd., Shenzhen, China) and the carbon content of the glucose monohydrate (36.36% w/w carbon, purity 100%; Xiwang Sugar Co., Ltd., Shandong, China). The glucose was mixed with the feed evenly before adding it to the tanks. During the 21-day experiment, no probiotics were used and no visible biofloc particles were observed in the water. All tanks were identically managed in terms of daily water exchange rate and feed schedule (Supplementary Fig. S7).

During the experiment, the water temperature, pH, dissolved oxygen and salinity were tracked daily at 7:00–8:00 am using a multiparameter water quality sonde (YSI Pro Plus, USA). One liter of water and five shrimps were collected from each tank weekly at approximately 9:00. Half of the water samples were used to analyze the total ammonia–nitrogen, nitrite-nitrogen, phosphate, total phosphorus and Chlorophyll *a* (Chl-*a*) according to the standard methods (AQSIQ 2007). The remaining water was sequentially filtered through 100 and 0.22 μm pore-size filters (Millipore, USA) to collect microbial biomass. Five guts were picked from the shrimp with a sterile toothpick and were pooled to compose one sample. All the microbiological samples were stored at −80 ℃ until DNA extraction. At the end of the experiment, 30 shrimps were randomly taken from each tank to measure individual length and weight, and all shrimps were harvested to calculate yield and survival rate.

### DNA extraction, 16S rRNA gene amplicon sequencing

Genomic DNA of water and gut samples were extracted using a Power Soil® DNA Isolation Kit (MoBio, USA), and a QIAamp® DNA Stool Mini Kit (Qiagen, Germany) according to the manufacturer’s instructions, respectively. Extracted DNA was assessed with a NanoDrop 2000 spectrophotometer (NanoDrop Technologies, Wilmington, DE, USA). The V4 variable regions of bacterial 16S rRNA gene were amplified using primers 515F-Y (5'-GTGYCAGCMGCCGCGGTAA-3') and 806R-B (5'-GGACTACNVGGGTWTCTAAT-3') (Apprill et al. 2015). Polymerase chain reaction (PCR) was performed in triplicate for each sample in a 20-μl reaction system under the following conditions: initial denaturation at 95 °C for 3 min; then 27 cycles of denaturation at 95 °C for 30 s, annealing at 55 °C for 30 s and extension at 72 °C for 45 s, with a final extension at 72 °C for 10 min. PCR products were quantified using an Agilent Bioanalyzer 2100 (Agilent, USA) with a Qubit fluorometer (Life Technologies, USA), and then sequenced on an Illumina MiSeq 2 × 250 bp platform (Illumina, USA).

### Processing of illumina sequencing data

Paired-end reads were joined with FLASH using default setting (Magoč and Salzberg 2011). The joined pairs were then processed with QIIME v 1.9.1 (Caporaso et al. 2010). Briefly, the reads were quality filtered using the *split_libraries_fastq.py* script at Q20 (Bokulich et al. 2013), and the chimeras in the quality-qualified sequences were identified with the USEARCH 6.1 (Edgar 2010). After removal of the chimeras, the remaining sequences were classified into operational taxonomic units (OTUs, 3% sequence cutoff) using the *pick_open_reference_otus.py* script with the Sortmerna_sumclust method (Kopylova et al. 2012). The representative OTU sequences were taxonomically assigned against SILVA128 database (Quast et al. 2012). OTUs affiliated with archaea, chloroplasts, mitochondria, unclassified (not affiliated with bacteria), as well as singleton OTUs were discarded. To normalize the differences in sequencing depth, the relative abundances of bacterial taxa in the bacterial communities were calculated based on OTU table rarefied at 18,000 reads per sample. A total of 3,941,538 high-quality reads were obtained, including 1,966,667 sequences of water samples (*n* = 57, 34,503 ± 724 per sample) and 1,974,871 sequences of gut samples (*n* = 57, 34,647 ± 704 per sample).

### Microbiome data analysis and statistics

The α-diversity indices (observed species, phylogenetic diversity and Shannon index), and β-diversity based on Bray–Curtis dissimilarity were calculated using QIIME 1.9.1. Pielou’s evenness was calculated using the ‘vegan’ package (Dixon 2003) in R environment. Bray–Curtis dissimilarity was used to compare the similarities of bacterial communities between/within groups. Principal Coordinates Analysis (PCoA) based on Bray–Curtis dissimilarity was applied to visualize the variation and dissimilarity in bacterial community structure between two groups. Permutational multivariate analysis of variance (PERMANOVA) (Anderson 2001) and analysis of similarity (ANOSIM) (Clarke 1993) were performed with the ‘vegan’ R package. Pearson’s correlation between the abundant OTUs and shrimp growth parameters, and Spearman correlation between *Ruegeria* and *Vibrio* OTUs were visualized using the ‘pheatmap’ R package (Kolde and Kolde 2015). The relative importance of the discriminatory OTUs on shrimp growth parameters was evaluated by boosted regression tree (BRT) analysis with the “gbm” package in R (Guo et al. 2017; Wang et al. 2017). BRT analysis is a machine-learning approach with advantages in dealing with non-linear relationships, and this complex nonlinear relationship can be reflected by the relative influence of each independent variable on response variables created during BRT calculation. In the present study, the growth performance parameters were selected as the response variable and the bacterial OTUs were used as predictor variables. Parameters including Gaussian error distribution, a learning rate of 0.01 and a bag fraction of 0.75 were set in BRT examination. Only predictor variables (OTUs) with relative importance greater than 2% are presented in the figure. The significance between two groups was determined by Independent *t*-test using SPSS 19.0. The data used for Independent *t*-test must meet normality and homogeneity of variance.

Interspecies interaction was evaluated by Molecular Ecological Network Analysis (MENA) using an open accessible pipeline (http://ieg2.ou.edu/MENA) (Deng et al. 2012). The top 200 abundant OTUs were used to build the MENA networks. Before network construction, random matrix theory (RMT) was applied to automatically identify the appropriate similarity threshold. In MENA networks, nodes representing OTUs and edges representing associations between OTUs. To quantitatively compare the differences in intestinal interspecies interaction between the control and glucose addition groups, multiple topology properties were calculated, including average degree (avgD), average path length (avgPL) and average clustering coefficient (avgCC). Networks were graphed using Cytoscape 3.6.1.

## Accession number of nucleotide sequences

All sequencing data generated in this study are publicly available from the Genome Sequence Archive in the BIG Data Center, Chinese Academy of Sciences (http://bigd.big.ac.cn/gsa, accession number: CRA003340).

## Supplementary Information

Below is the link to the electronic supplementary material.

Supplementary file1 (DOCX 3929 KB)
