# Supplementary material for: Contrasting patterns of bacterial communities in the rearing water and gut of Penaeus vannamei in response to exogenous glucose addition
Source: Mar Life Sci Technol. 2022 Jan 1;4(2):222–36. doi: 10.1007/s42995-021-00124-9 (PMC10077327; doi:10.1007/s42995-021-00124-9)
Supplement: Supplementary file 1 — Supplementary file1 (DOCX 3929 KB) [file 42995_2021_124_MOESM1_ESM.docx]

**Supplemental Data Summary**

**Contrasting patterns of** **bacterial communities in the rearing water and gut of *Penaeus vannamei* in response to exogenous glucose addition**

Lei Huang^1,2,3^, Haipeng Guo^1,2^*, Zidan Liu^1,2^, Chen Chen^4^, Kai Wang^1,2^, Xiaolin Huang^2,4^, Wei Chen^1,2^, Yueyue Zhu^1,2^, Mengchen Yan^1,2^, Demin Zhang^1,2^*

^1^ State key laboratory for managing biotic and chemical threats to the quality and safety of agro-products, Ningbo University, Ningbo 315211, China

^2^ School of Marine Sciences, Ningbo University, Ningbo 315211, China

^3^ Zhejiang Institute of Freshwater Fisheries, Huzhou 313001, China

^4^ Zhejiang Mariculture Research Institute, Wenzhou 325005, China

Corresponding authors:

*E-mail [guohaipeng@nbu.edu.cn](mailto:guohaipeng@nbu.edu.cn); Tel. (+86) 150 8862 6765; Fax (+86) 0574 8760 0164.

*E-mail [zhangdemin@nbu.edu.cn](mailto:zhangdemin@nbu.edu.cn); Tel. (+86) 135 6743 4998; Fax (+86) 0574 8760 0164.





**Fig. S1** Temporal dynamics of water physicochemical parameters in the control and glucose groups. Data present means ± standard error (n = 8). The statistical significance of the differences between the two groups are tested using an independent *t*-test (* *P* < 0.05, ** *P* < 0.01).





**Fig. S2** Growth performance of *Penaeus vannamei* in the control and glucose groups at the end of the 21-day experiment. Data present means ± standard errors (n = 8). The statistical significance of the differences between the two groups are tested using an independent *t*-test (** *P* < 0.01).





**Fig. S3** Relative abundances of the dominant phyla/proteobacterial classes with (average relative abundance > 1%) and families (> 3%) in the rearing water (**A**) and shrimp guts (**B**). C: control group; G: glucose group.





**Fig. S4 A** Heatmaps illustrate the relationship (Pearson’ correlation) between the discriminatory OTUs of rearing water (from Figure 4) and shrimp growth parameters at day 21. * and ** represent statistical significance at *P* < 0.05 and *P* < 0.01 levels, respectively. **B** The relative importance of these discriminatory OTUs in the rearing water on shrimp growth parameters in the BRT model. OTUs with relative importance greater than 2% are displayed in the figure.





**Fig. S5** Networks and related topological properties of the control and glucose groups in the rearing water. The color of the node shows different dominant classes, and the relative abundances of these classes are showed in the bar charts. The color of the edge shows positive (green) or negative (red) correlations between nodes. Rhodobacteraceae (blue circles) is separately grouped in each network. avgK: average degree; avgCC: average clustering coefficient; GD: average path distance.


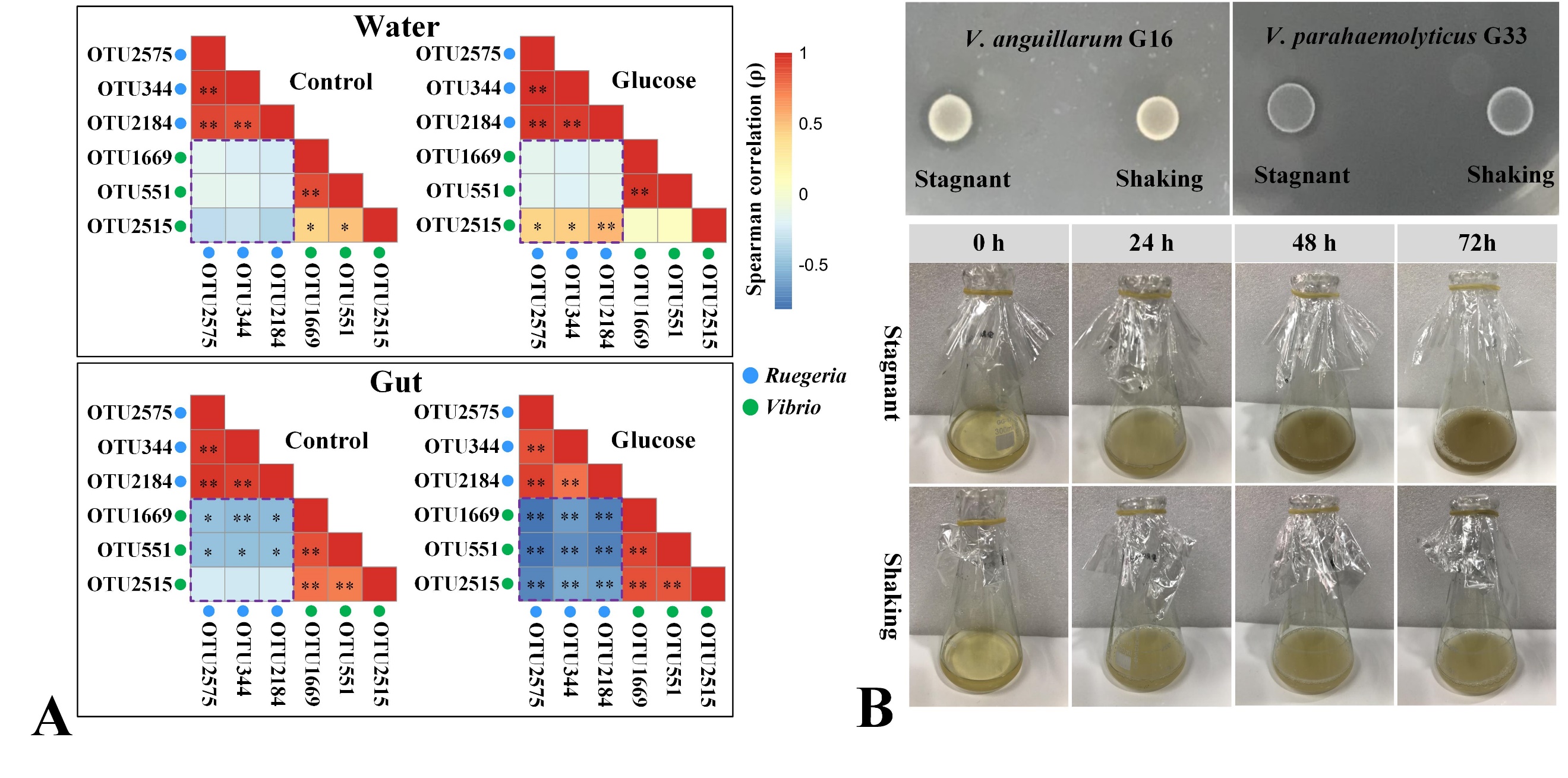


**Fig. S6 A** Heatmaps illustrate the relationship (Spearman correlation) between the abundant (relative abundance > 0.1%) *Rugeria* OTUs and *Vibrio* OTUs at the control and glucose groups in rearing water and shrimp gut. * and ** represent statistical significance at *P* < 0.05 and *P* < 0.01 levels, respectively. **B** The upper figures show that the antimicrobial activity assay of *Ruegeria* sp. G36 against *Vibrio* isolates under stagnant or shaking culture conditions after 72 hours of incubation. The below figures show that the color change of the culture medium of *Ruegeria* sp. G36 during 72 hours of incubation under stagnant or shaking culture conditions.





**Fig. S7** Timeline showing the sampling schedules and important husbandry events over the course of the experiment.

**Table S1** The nodes and edges associated with Rhodobacteraceae in the bacterial co-occurrence networks of rearing water and shrimp gut.

|  |  | Water | |  | Gut | |
| --- | --- | --- | --- | --- | --- | --- |
|  |  | Control | Glucose |  | Control | Glucose |
| Total nodes of network |  | 127 | 110 |  | 131 | 143 |
| Rhodobacteraceae nodes |  | 18  (14.2%) | 33  (30.0%) |  | 33  (25.2%) | 53  (37.1%) |
| Total edges of network |  | 209 | 173 |  | 181 | 293 |
| Rhodobacteraceae associated edges |  | 27  (12.9%) | 86  (49.7%) |  | 76  (42.0%) | 170  (58.0%) |
